# Effect of the dietary supplement PERMEAPROTECT+ TOLERANCE© on gut permeability in a human co-culture epithelial and immune cells model

**DOI:** 10.1016/j.heliyon.2024.e28320

**Published:** 2024-03-27

**Authors:** Anne Abot, Nicolas Pomié, Gwendoline Astre, Patrice D. Cani, Justine Aussant, Emmanuel Barrat, Claude Knauf

**Affiliations:** a Enterosys SAS, 31670 Labège, France; b Metabolism and Nutrition Research Group, Louvain Drug Research Institute LDRI, UCLouvain, Université catholique de Louvain, Brussels, Belgium; c WELBIO-Walloon Excellence in Life Sciences and Biotechnology, WELBIO department, WEL Research Institute, avenue Pasteur, 6, 1300, Wavre, Belgium; d NeuroMicrobiota, International Research Program (IRP) INSERM/UCLouvain, France Belgium; e UCLouvain, Université catholique de Louvain, Institute of Experimental and Clinical Research IREC, 1200, Brussels, Belgium; f Laboratoire Lescuyer, Research Department, 15 rue Le Corbusier, 17440, Aytré, France; g INSERM U1220, Institut de Recherche en Santé Digestive IRSD, Université Paul Sabatier, Toulouse III, CHU Purpan, Place Du Docteur Baylac, CS 60039, CEDEX 3, 31024 Toulouse, France

**Keywords:** Intestinal permeability, Epithelial cells, Immune cells, Antioxidant

## Abstract

**Background and objective:**

The leaky gut syndrome is characterized by an intestinal hyperpermeability observed in multiple chronic disorders. Alterations of the gut barrier are associated with translocation of bacterial components increasing inflammation, oxidative stress and eventually dysfunctions of cellular interactions at the origin pathologies. Therapeutic and/or preventive approaches have to focus on the identification of novel targets to improve gut homeostasis. In this context, this study aims to identify the role of PERMEAPROTECT + TOLERANE©, known as PERMEA, a food complement composed of a combination of factors (including l-Glutamine) known to improve gut physiology.

**Methods:**

We tested the effects of PERMEA or l-Glutamine alone (as reference) on gut permeability (FITC dextran method, expression of tight junctions) and its inflammatory/oxidative consequences (cytokines and redox assays, RT-qPCR) in a co-culture of human cells (peripheral blood mononuclear cells and intestinal epithelial cells) challenged with TNFα.

**Results:**

PERMEA prevented intestinal hyperpermeability induced by inflammation. This was linked with its antioxidant and immunomodulatory properties showing a better efficacity than l-Glutamine alone on several parameters including permeability, global antioxidant charge and production of cytokines.

**Conclusion:**

PERMEA is more efficient to restore intestinal physiology, reinforcing the concept that combination of food constituents could be used to prevent the development of numerous diseases.

## 1 Introduction

The intestinal epithelium is a permeable barrier that plays a major role in the regulation of solute and fluid exchanges and has a direct impact on nutrient absorption and transport. This epithelium is composed of several cell types: enterocytes, goblet cells, M cells, Paneth cells and others, such as endocrine and immune cells. The cooperation between all these cells maintains the integrity of the mucosa, which is challenged daily by external factors in the luminal environment. Oxidative stress and inflammation are known to participate to the alteration of the gut barrier, but the etiology of intestinal inflammation, which is increasing worldwide, is not yet fully understood, although multiple causes are known to be involved including infection, dietary habits (e.g. high-fat diet, dietary emulsifier), stress reaction, and the composition of the gut microbiota [ [1,2]]. Consequently, low- or high-grade intestinal inflammation are often associated with different pathologies such as chronic diarrhea, abdominal pain, inflammatory bowel diseases, neurodegenerative disorders but also metabolic diseases (e.g. obesity, type 2 diabetes) [ [3,4]].

Reducing the disruption of this intestinal barrier referred as the leaky gut is proposed as future preventive and/or therapeutic option to treat gut associated pathologies [5]. The intestinal epithelium is widely exposed to food components and toxins, including bioactive compounds with potential health risks or benefits. As example, phytochemicals such as polyphenols or carotenoids have been shown to have anti-inflammatory and antioxidant effects and benefits for chronic disorders [6]. Among all food complements, glutamine is considered as one major compound that improves intestinal permeability by targeting tight junctions, oxidative stress and inflammation [7]. Moreover, recent evidence suggests several dietary nutrients are involved in the regulation of intestinal permeability [8]. Furthermore, the use of combination of glutamine and other bioactive substances have been suggested to reduce intestinal hyper-permeability in irritable bowel syndrome [9].

In recent decades, *in vitro*, *ex vivo* and *in vivo* models have been developed to study different functions and metabolism of the intestinal epithelium, especially in proinflammatory condition. Although animal models allow the study of the integrated physiology of a whole organism, the main drawbacks of *in vivo* models are the variation in responses due to species differences and the difficulty of extrapolating results to humans [10]. Intestinal epithelial cells (IEC) culture models have been widely used in bioavailability and toxicology studies in the food and pharmaceutical fields. Improved *in vitro* models using cells of human origin have the advantage of limiting the use of animal experiments and allows the study of the molecular mechanisms of a therapeutic compound in a simple and reproducible manner [11].

The human colon cell line HT-29 is not only used to study human colon cancers, but it is receiving special interest in studies focused on food digestion and bioavailability due to the ability to express characteristics of mature intestinal cells. Specific external and internal signals, particularly the presence of microbiota, shape these cells to better cooperate with the gut ecosystem, controlling intestinal physiology. The integrity of intestinal epithelial barrier represents a key feature of gut immune tolerance, which can be regulated by multiple factors. Several evidences describe close interactions between peripheral blood mononuclear cells (PBMC) and gut epithelium that significantly contribute to intestinal barrier function [12]. This interaction involves a complex dialogue, where PBMCs contribute to the regulation of the barrier function through cytokine secretion, influencing the epithelial cells' response to inflammation and stress [[13], [14], [15], [16]]. The alteration of this dialogue results in a modified secretion of pro- and anti-inflammatory cytokines. Moreover, drugs or bioactive compounds with an anti-inflammatory effect such as butyrate or 1α,25-dihydroxyvitamin D3 may have a beneficial effect in restoring functional communication between these two cell types [ [12,13]].

Here, the aim of this study was to evaluate the potential protective impact of PERMEAPROTECT + TOLERANCE (also known as PERMEA), a glutamine-rich dietary supplement on the French market (that also contains palmitoylethanolamine, polyphenols, β-cartonene, zinc and vitamins B1, B3 and D3), on intestinal physiology (gut permeability, anti-inflammatory and anti-oxidative effects) in a transwell co-culture model favorizing the functional dialogue between IEC and PBMC cells. We also compared its effects to those of glutamine alone.

## 2 Methods

### 2.1 PBMC cells

To fulfill our objectives, n = 5 donors and 2 assays per condition were required. The 5 different donors were male, between 18 and 40 years-old, from Caucasian origin, non-smoker and with a BMI between 18 and 25 (Supplemental Table S1).

### 2.2 Cell culture

HT-29 cell line were cultured at 37 °C in a humidified 5% CO_2_ incubator in Dulbecco modified eagle medium (DMEM) with high concentration of glucose, supplemented with 10% heat-inactivated fetal bovine serun (FBS), 3.97 mM L-alanyl-glutamine and 1% Penicillin-Streptomycin (P/S). The cell line adheres to the flasks and were therefore removed using trypsin/EDTA solution. The media was changed every day to maintain a high glucose concentration. Prior to the experiments, HT-29 cells were seeded 0.4 μm, 1 cm^2^ transwell™ filters and grown to confluence, then deprived in glutamine for 48h. The deprivation of HT-29 cells in glutamine for 48 h was a preparatory step to create a controlled environment for assessing the effects of glutamine and PERMEA on the cells under inflammatory conditions.

PBMC (2 × 10^6^ cells/ml) were plated in duplicate in a 24-well culture plate in RPMI 1640 medium supplemented with 10% heat-inactivated FBS, 1% l-glutamine (2 mM) and 1% Penicillin-Streptomycin (P/S) at 37 °C in a humidified chamber with 5% CO_2_.

### 2.3 Co-culture model

Following a 24-h recovery period for the PBMC, co-culture with HT-29 was initiated. The basolateral side of the HT-29 monolayers, deprived of glutamine and established on Transwell™ inserts, was brought into contact with 0.5 mL of PBMCs (2 × 10^6^ cells/mL) cultures. These co-cultures were maintained for 24 h at 37 °C with 5% CO_2_, in media with or without the compounds of interest, i.e., l-glutamine (8.5 mM, used as a reference), or varying concentrations of PERMEA.

The co-cultures were then subjected to stimulation with TNF-α (200 ng/mL in the basolateral medium) for 6 h, using fresh cell media with or without the compounds of interest. After the treatments, the supernatants and cell pellets were stored at −80 °C until further analysis.

The co-culture model of HT-29 cells and PBMCs provides a particularly relevant platform for studying the effects of molecules of interest on the digestive sphere. HT-29 cells, being human colon adenocarcinoma cells, represent the epithelial layer of the intestinal barrier, pivotal in nutrient absorption and barrier functions. On the other hand, PBMCs encompass various immune cell subsets, reflecting the complex immunological environment of the gut. By co-culturing these cells, we can mimic the intricate interactions between the epithelial barrier and the immune system – a key feature of the gastrointestinal environment. Additionally, we can simulate the disruption of the intestinal barrier by stimulating PBMCs with TNF-α, thereby inducing an inflammatory response. This response has been previously characterized (unpublished data) by an increased production of the proinflammatory cytokine interleukin 8 (IL-8) by PBMCs, along with an increase in transepithelial permeability to macromolecules. This makes it an invaluable model for understanding how target molecules influence not only epithelial functions but also modulate local immune responses, encapsulating a more holistic view of their roles in digestive health. Consequently, the co-culture model presented here allows us to examine overall reactivity while maintaining cell interconnections as closely as possible to physiological conditions. As a result, the data obtained reflect the comprehensive effects of the tested molecules.

### 2.4 Preparation of PERMEA solution and treatments

PERMEAPROTECT + TOLERANCE (PERMEA, Laboratoire Lescuyer, Aytré, France), the tested product, is a food supplement commercially available in France. It is used to help preserving the intestinal mucosae, mostly in case of minor gut inflammation accompanying irritable bowel syndrome, allergy or metabolic syndrome. PERMEA is composed of several ingredients including maltodextrin, l-glutamine, natural flavors, palmitoylethanolamide, grape-seed extract (Vitis vinifera L.) titrated in polyphenols, sodium citrate, zinc gluconate, vitamins (beta-carotene, D3, thiamine, folic acid) and, steviol glycosides. In its commercial form, the composition in actives of one 5-gramms stick is: l-glutamine 1.5g, palmitoylethanolamide 150 mg, grape-seed extract 135 mg including polyphenols 120 mg, vitamin A (beta-caroten) 400 μg ER, vitamin D3 10 μg, thiamine (vitamin B1) 0.7 mg, folic acid (vitamin B9) 150 μg, zinc 7.5 mg. The effect of PERMA was investigated using the soluble fraction of the product. First, the content of three 5-gramms sticks of the commercially available product was grinded with a porcelain pestle and mortar. Then, the powder obtained was dissolved in the appropriate volume of sterile water. After 1 h of shaking at 37 °C, the insoluble fraction of the solution was sedimented. The maximal volume of the soluble fraction was collected. The insoluble fraction was then dissolved in 1% EtOH and 1% DMSO after 1 h of shaking at 37 °C. Finally, the two fractions were pooled, centrifuged for 5 min at 4000g to remove the insoluble fraction (mostly composed of the carotene encapsulation residues) and 0.22 μm filtered. The concentration of PERMEA solutions were expressed in g/l corresponding to the mass concentration of the commercially available product. The actives’ concentrations of the tested solutions are shown in Table 1.

**Table 1:** Concentration of actives of the tested solutions.

	TESTED SOLUTIONS		
	l-Glutamine	PERMEA (2.1 g/L)	PERMEA (4.2 g/L)
l-glutamine (mM)	8.6	4.3	8.6
Palmitoylethanolamide (PEA, mM)		0.2	0.4
Grape polyphenols (eq. Gallic acid, mM)		0.3	0.6
β-carotene (eq. Retinoic acid, μM)		0.6	1.2
Vitamin D3 (nM)		10.8	21.7
Thiamine (vitamin B1, μM)		1.1	2.2
Folic acid (vitamin B9, μM)		0.15	0.30
Zinc (μM)		47.8	95.6

### 2.5 Cell viability

The measurement of lactate dehydrogenase activity in the extracellular medium (LDH assay) associated to an indicator of cellular metabolic activity of cells (MTT assay) were used on 2D monolayer culture. To evaluate the toxicity of the tested items on IEC, the cells were treated with 5 concentrations of tested item (D1 = 33.3 g/l; D2 = 8.3 g/l; D3 = 2.1 g/l; D4 = 0.52 g/l; D5 = 0.13 g/l) for 24h. Then, the supernatant was collected at the end of the treatment period. The activity of the lactate dehydrogenase released by damaged cells was quantified using the LDH assay. The viability of IEC was also evaluated by measuring the formazan produced by metabolic active cells using the MTT cell proliferation assay. The combination of these two cell viability tests allowed us to determine the two working concentrations of tested item.

### 2.6 Gene expression

The RNA of the cells was extracted individually using TriReagent, according to the manufacturer's instructions. Quantification analysis of total RNA was performed by analyzing 1 μl of each sample in Nanodrop. cDNA was prepared by reverse transcription of 500 ng total RNA using a RT iScript kit (Biorad). Real-time PCR was performed with the Via7 real-time PCR system and software (Applied biotechnology) using SYBR Green Real-Time PCR Master Mixes (Biorad) for detection, according to the manufacturer's instructions. *Gapdh* was chosen as the housekeeping gene [17]. All samples were performed in duplicate, and data were analyzed according to the 2^ΔΔCT^ method. The identity and purity of the amplified product were assessed by melting curve analysis at the end of amplification. The IEC expression of the genes encoding for the following proteins were analyzed: zonula occulens 1 (*Zo-1)*, claudin 4 (*Cldn4)*, occludin (*Ocln)*, defensin β1 (*Defb1)*, nuclear factor erythroid 2-related factor 2 (*Nrf-2)* and mucin 2 (*Muc2)* (Table 2).

**Table 2:** Primers sequences.

Gene	Sens	Antisens
*Gapdh*	GCCACATCGCTCAGACACCAT	CAGGCGCCCAATACGACCAAA
*Nrf2*	TTTTCCCCAGAGCGGCTTTGTC	GCCTCCAAAGTATGTCAATCAAATCC
*Cldn4*	AAGGTGTACGACTCGCTGCTG	ATGCTGATGATGACGAGGGCG
*Ocln*	AGAAAGTGAAGAGTACATGGCTGCT	CTCCAACCATCTTCTTGATGTGTGA
*Zo-1*	AAGATGTCCGCCAGAGCTGC	GCGTCACTGTATGTTGTTCCCA
*Muc2*	CATCGAGTGCAGGTCGGTCAA	CACACTGCACCTTCTGGCCTA
*Defb1*	TGCCAGTCGCCATGAGAACTT	GACATTGCCCTCCACTGCTGA

### 2.7 Permeability assay

Paracellular permeability was assessed after treatment on another cell coculture. The cells were gently rinsed with DPBS and incubated in the apical side with Hank's balanced salt solution (HBSS) containing 1 mg/mL FITC-dextran (4.4 kDa) solution for 1 h. FITC-dextran flux was assessed by taking 100 μL from the basolateral chamber. Fluorescent signal was measured with a microplate reader using 492 nm excitation and 520 nm emission filters. FITC-dextran concentrations were determined using standard curves generated by serial dilutions. The concentration of FITC-dextran from the basolateral side represents the severity of permeability defect of HT-29 cells.

### 2.8 Elisa assay

The apical and basolateral supernatants were clarified by centrifugation (1000 g 20 min 4 °C). The supernatants were stored at −80 °C and were used for evaluating the immunomodulatory properties of the tested conditions using IL-1β and IL-8 (for the apical side) and IL-1β, IL-10 and IL-12 (for the basolateral side) ELISA kits (Merck, Germany). Measurements were assessed according to the manufacturer instructions.

### 2.9 Antioxidant capacity

The antioxidative capacities of l-Glutamine and PERMEA were evaluated in the apical cell culture media after a 24h incubation time with e-BQC reader (Libios, France). The measure is based in a redox potential measure, its charge referred to the antioxidant capacity. e-BQC differentiated the fast and slow acting antioxidants (for example ascorbic acid *vs* polyphenols). These two values of charge were: Q1 (fast antioxidants), referred to the antioxidant capacity of the compounds with the highest rate of free radical scavenging; Q2 (slow antioxidant), referred to the antioxidant capacity of the compounds with a lower rate of free radical scavenging. Briefly, 60 μl of the apical supernatants were collected and used for the assessment of total antioxidant capacity. The results were given in a charge unit (μC). Those were representing the charge of the electrons that were released by the antioxidants present in the biological cell media to neutralize free radicals.

### 2.10 Data analysis

The data were expressed as the mean ± SEM. Differences between the experimental groups were assessed where appropriate using by unpaired Student's, one- or two-way ANOVA, followed by post-hoc test. Data were analyzed using GraphPad Prism version 8.00 for Windows (GraphPad Software, San Diego, CA, USA). The results were considered statistically significant at p < 0.05.

## 3 Results

### 3.1 Cytotoxicity and viability of the tested items on IEC

We considered that when the experimental condition exceeds the threshold of 20% of Triton lysis control, a potential cytotoxic effect occurred. We observed a slight increase in LDH activity with the concentration of PERMEA 8.3 g/L compared to the control condition and an increase over 20% cytotoxic threshold in response to PERMEA 33.3 g/L (Supplemental Fig. S1).

The viability of cells treated by tested items was evaluated by the formazan production measurement. The results were represented in percentage of the control condition without l-Glutamine. We considered that when the percentage is between 80 and 120%, no potential cytotoxic effect occurred. First, we observed that the higher concentrations of PERMEA (i.e., 0.25 and 1%) negatively impacted the proliferation rate measured by MTT assay. Furthermore, we noted that l-Glutamine and PERMEA were able to protect from this deleterious effect with a slight increase of proliferation process. This effect was dose-dependent in response to PERMEA solution (Supplemental Fig. S2).

To summarize, we did not observe any modifications of cytotoxicity or loss of cell viability in response to l-Glutamine, regardless of the dose used. However, in response to higher doses of PERMEA (8.3 and 33.3 g/L), we showed higher LDH activity. Accordingly, we used the working concentrations of PERMEA 2.1 and 4.2 g/L and the concentration of l-Glutamine 8.6 mM (corresponding to the same concentration found in 4.2 g/L of PERMEA, see Supplemental Table S2) for the next steps. The concentrations of PERMEA were representative of half and a quarter of those found in an empty stomach.

### 3.2 Intestinal permeability and barrier function

**Fig. 1:**
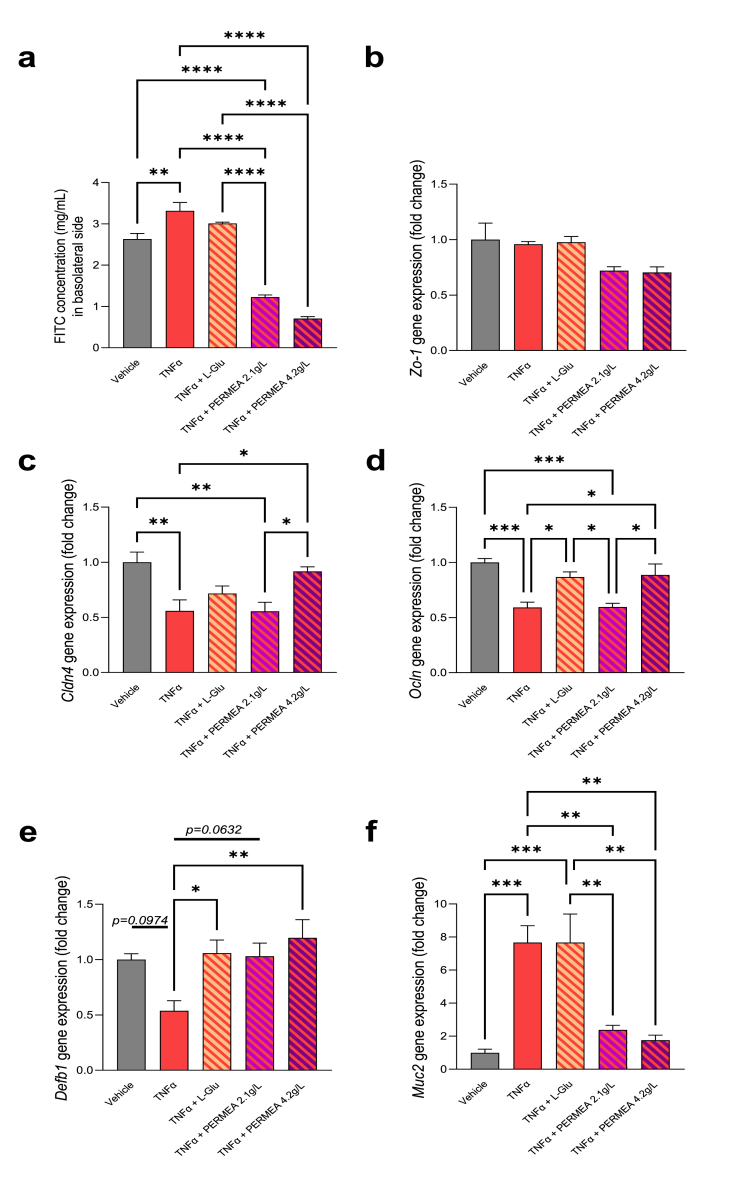
**Intestinal permeability and barrier function properties of PERMEA and****l****-glutamine (L-Glu) in an IEC/PBMC co-culture model, in response to exposure to TNFα. (a)** Permeability of IEC monolayer measured by transfer of fluorescent dextran (4,4 KDa), **p < 0.01 *vs* Vehicle, ****p < 0.0001 *vs* Vehicle, *vs* TNFα or *vs* TNFα + L-Glu, **(b)***Zo-*1 mRNA expression was quantified using RT-qPCR in IEC, no significant difference was observed **(c)***Cldn4* mRNA expression was quantified using RT-qPCR in IEC, *p < 0.05, **p < 0.01 *vs* Vehicle, *vs* TNFα or *vs* TNFα + PERMEA, **(d)***Ocln* mRNA expression was quantified using RT-qPCR in IEC, *p < 0.05, ***p < 0.001 *vs* Vehicle, *vs* TNFα, *vs* TNFα + L-Glu or *vs* TNFα + PERMEA, **(e)***Defb1* mRNA expression was quantified using RT-qPCR in IEC, *p < 0.05, **p < 0.01 *vs* TNFα, **(f)***Muc2* mRNA expression was quantified using RT-qPCR in IEC, **p < 0.05, ***p < 0.001 *vs* Vehicle, *vs* TNFα or *vs* TNFα + L-Glu. The associated p-values were obtained using 1-way ANOVA followed by Bonferroni's post-hoc test. n = 5/group.

In a proinflammatory environment induced by TNFα, we observed an increase in the transfer of FITC-dextran in basolateral side reflecting altered integrity of IEC tight junctions (Fig. 1a). Unlike l-Glutamine, PERMEA was able to reverse the deleterious effect induced by TNFα regardless the tested concentration. These results were correlated with the restoration of the mRNA expression of tight junctions claudin-4 (*Clnd4*) and occludin (*Ocln*) in response to PERMEA, without impact on zonula occludens-1 (*Zo-1*) mRNA expression (Fig. 1b–d). l-Glutamine restored only *Ocln* mRNA expression.

Β-Defensin 1 is an antimicrobial peptide produced by colonic epithelia and constitutively expressed. In the intestinal tract, this biomarker contributes to host immunity and assists in maintaining the balance between protection from pathogens [18]. In response to TNFα, *Defb1* mRNA expression decreased by 50% (p = 0.09), but was completely normalized with l-Glutamine or PERMEA treatment with the same efficacy (Fig. 1e). The mRNA expression of *Muc2* coding for the oligomeric mucus gel-forming mucin 2, the main component of the mucous barrier that serves to protect the colonic epithelium, was drastically increased by inflammation induced by TNFα. The PERMEA treatment prevented this effect regardless of the dose tested, while l-Glutamine alone did not improve this parameter (Fig. 1f).

### 3.3 Immune modulation capacities

**Fig. 2:**
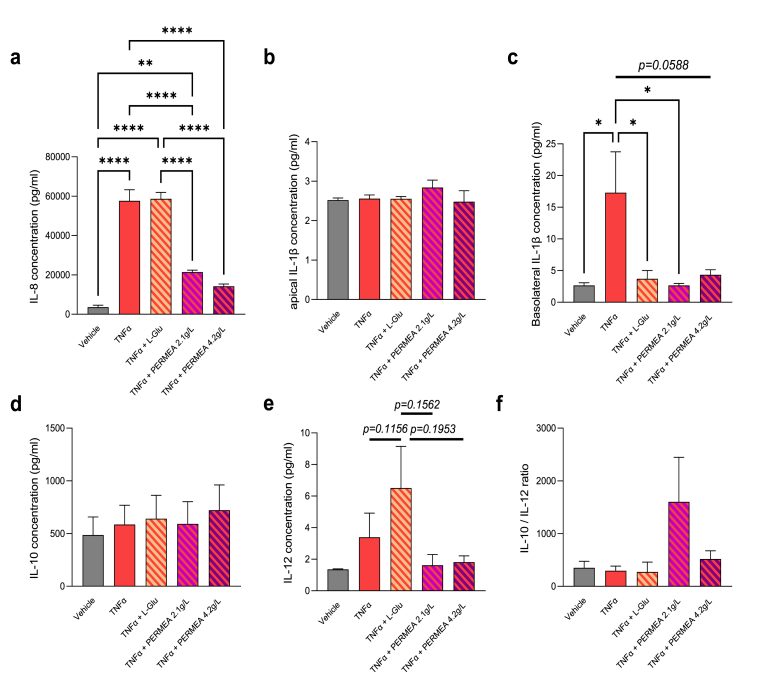
**Immunomodulation properties of PERMEA in an IEC/PBMC co-culture model.** The cytokines release of each experimental condition was evaluated using ELISA assays after a treatment with vehicle, TNFα with or without l-Glutamine (L-Glu) or PERMEA. **(a)** IL-8 secretion was quantified in the apical cell culture media, **p < 0.01 *vs* Vehicle, ****p < 0.0001 *vs* Vehicle, *vs* TNFα or *vs* TNFα + L-Glu, **(b)** IL-1β secretion was quantified in the apical cell culture media, no significant difference was observed, **(c)** IL-1β secretion was quantified in the basolateral cell culture media, *p < 0.05 *vs* TNFα, **(d)** IL-10 secretion was quantified in the apical cell culture media, no significant difference was observed, **(e)** IL-12 secretion was quantified in the apical cell culture media, no significant difference was observed, **(f)** IL-10/IL-12 ratio has been calculated. The associated p-values were obtained using 1-way ANOVA followed by Bonferroni's post-hoc test. n = 5/group.

The quantification of the concentration of pro-inflammatory and anti-inflammatory cytokines was performed in apical side (i.e., cytokines released by IEC) and in basolateral side (i.e., cytokines released by IEC and/or PBMC) in response to vehicle, TNFα or the solutions of l-Glutamine or PERMEA (Fig. 2).

In the apical compartment, we observed that TNFα-induced stress strongly enhanced the secretion of the pro-inflammatory cytokine IL-8, whereas the two tested concentrations of PERMEA markedly decreased IL-8 levels. l-Glutamine had no effect on this parameter (Fig. 2a). We did not observe any variation of IL-1β in the apical side for the different experimental conditions. Conversely, IL1-β level increased in basolateral in response to TNFα and this effect was counteracted by l-Glutamine and PERMEA (Fig. 2b and c).

We measured on the basolateral compartment the secretion of IL-10 and IL-12 by the PBMC and/or IEC cells during the co-culture. As shown in Fig. 2d and e, the levels of IL-10 were not affected in the different experimental conditions, whereas the levels of IL-12 were enhanced in response to TNFα alone or in co-treatment with l-Glutamine, but this effect was non statistically significant. IL-12 levels did not increase in response to PERMEA, however, the interindividual variability prevented from concluding definitively, the same observation is true also for the IL-10/IL-12 ratio (Fig. 2f).

### 3.4 Antioxidant capacities

**Fig. 3:**
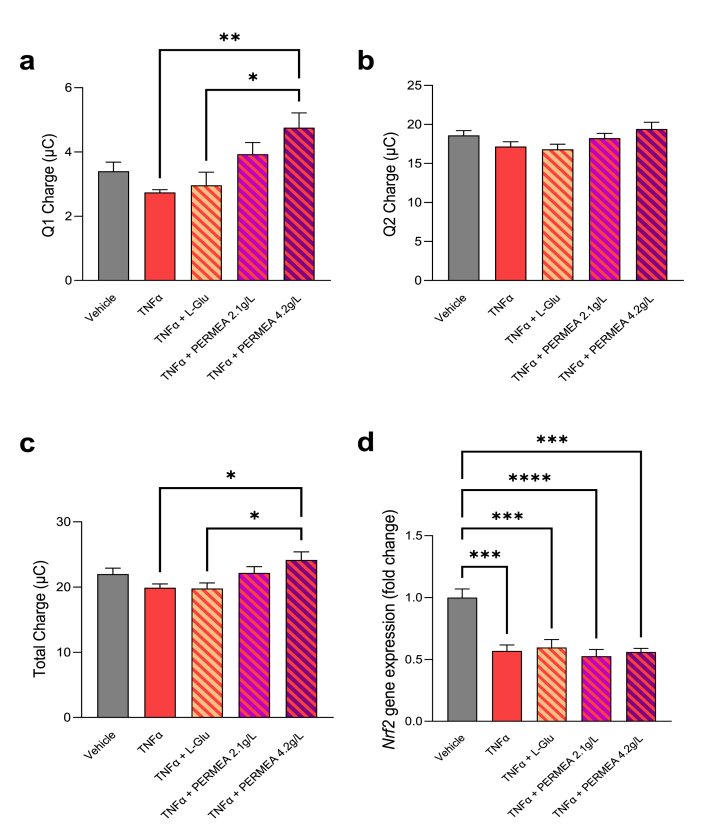
**Antioxidant capacities of PERMEA in an IEC/PBMC co-culture model.** The redox potential measure of each experimental condition was evaluated with e-BQC reader in the apical cell culture media, after a treatment with vehicle, TNFα with or without l-Glutamine (L-Glu) or PERMEA. **(a)** Fast antioxidant capacity, *p < 0.05, **p < 0.01 *vs* TNFα or TNFα + L-Glu. **(b)** Slow antioxidant capacity, no significant difference was observed. **(c)** Total antioxidant capacity, *p < 0.05 *vs* TNFα or TNFα + L-Glu. **(d)***Nrf2* mRNA expression relative expression, ***p < 0.001, ****p < 0.0001 *vs* Vehicle. In panel a, c and d, the associated p-values were obtained using 1-way ANOVA followed by Bonferroni's post-hoc test. n = 5/group.

We observed that the higher dose of PERMEA presented antioxidative capacities with a better efficacy than l-Glutamine, especially concerning fast antioxidants (Fig. 3a–c). Then, in IEC, the mRNA expression of *Nrf2*, a biomarker involved in the regulation of the physiological and pathophysiological outcomes of oxidant exposure, was quantified. The oxidative stress induced by TNFα decreased the mRNA expression of *Nrf2* but neither l-Glutamine nor PERMEA restored its physiological mRNA expression (Fig. 3d).

## 4 Discussion

PERMEA is a food supplement composed of l-glutamine, palmitoylethanolamine, polyphenols, vitamins and minerals. The first aim of this study was to evaluate the ability of PERMEA to prevent an increase in intestinal permeability induced by inflammation, as well as its antioxidant and immunomodulatory properties. To this aim we studied the impact of PERMEA on intestinal homeostasis by measuring the transepithelial flux of dextran, the global antioxidant charge, the mRNA expression of biomarkers, and the production of pro- and anti-inflammatory cytokines released from IEC or PBMC stressed with TNFα. The second objective was to compare the results in response to PERMEA *vs* l-Glutamine alone (at the same concentration of the higher dose of PERMEA) to discriminate potential additive effects of PERMEA components.

The integrity of intestinal epithelial barrier represents a key feature of gut immune tolerance, which can be regulated by multiple factors. Number of studies have demonstrated that TNFα disturbs tight junction expression and intestinal mucosa structure [19]. We demonstrated that PERMEA had a high efficacy on this parameter, in a dose dependent manner. Indeed, at the concentration of 2.1 g/L, we observe a beneficial impact only in FITC assay whereas at the concentration of 4.2 g/L, our data showed a decreased transfer of FITC-Dextran and a restoration of physiological mRNA expression of tight junctions (*Cldn4* and *Ocln*). Furthermore, l-Glutamine were only able to restore *Ocln* mRNA expression. These results highlight an effect of PERMEA components to maintain intestinal permeability reinforcing the importance of the use of a combination of dietary substances to treat pathologies. However, due to the lack of sufficient material, the present study focused mainly on mRNA expression, and we did not measure variations of proteins of tight junctions. Indeed, investigating protein expressions can be done by using the distribution of the tight junctions according to their solubility profiles. This requires using two different methods of extractions, that is one using proteins extracted from insoluble fraction and a second from soluble fraction. When proteins are delocalized from the junction protein complex associated with the cytoskeleton they are found in the soluble fraction leading to a possible altered gut barrier function [20]. Therefore, when the proteins are distributed to the insoluble fraction they are considered as correctly localized, when they are found also in the soluble fraction, this suggest a shift of intestinal junctional protein from the cytoskeleton, causing a decrease in paracellular sealing.

Several evidences describe close interactions between PBMC and gut epithelium that significantly contribute to the intestinal barrier function [12]. The alteration of this dialogue results in a modified secretion of pro- and anti-inflammatory cytokines. With the same efficacy, l-Glutamine and the higher concentration of PERMEA were able to restore a physiological mRNA expression of *Defb1*. Attenuated expression of defensins compromises host immunity and hence may alter the balance toward inflammation. Altered defensin production is suggested to be an integral element in the pathogenesis of inflammatory bowel disease [21]. Furthermore, we observed that the increase of *Muc2* mRNA expression induced by TNFα was only attenuated in response to PERMEA. This mechanism of TNFα action was previously described, involving the MAP Kinase pathway, and argues for a significant relationship between colonic carcinogenesis and Muc2 expression [ [22,23]]. Again, the present results also show an effect of PERMEA components *vs* l-Glutamine alone.

In this study, the results showed that PERMEA exerted anti-inflammatory properties by limiting the release of pro-inflammatory cytokines in apical side (IL-8) and in basolateral side (IL-1β and IL-12, in a non-significant manner) in response to TNFα. These beneficial effects were similar in response to the 2 concentrations of PERMEA tested but not observed in response to l-Glutamine alone, excepted for IL-1β release. These results suggest an impact of the combination of the different components present in the PERMEA solution which are already known to have an effect on inflammation, as is the case with vitamin D3 [24], palmitoylethanolamide [25], vitamin A, vitamin B9 and zinc [12]. The balance between the secretion of IL-10 and IL-12 in immune models has been an attractive parameter for the determination of the immune modulatory properties of compounds of interest [23,26]. It is well established that IL-12 plays a significant role in the activation of the Th1 immune response and, at the same time, that IL-10 action results in a negative regulation of this process. The apparent opposing relationship that exists between the two interleukins has led to several studies successfully employing the ratio of these cytokines (usually IL-10/IL-12) in the assessment of immune status, e.g., as a measure of the balance between an anti-inflammatory and a proinflammatory state, or Th1 *vs* Th2 dominance [ [26,27]]. This ratio tends to increase in response to PERMEA without reaching significance likely because of high inter-individual variability, in particular for one donor (i.e. donor 4) who secreted very high cytokine levels in response to TNFα.

Finally, our data showed that PERMEA exerted a significant antioxidative effect in the co-culture model by measuring the redox potential, although no effect was seen on *Nrf2* mRNA expression. PERMEA seems to be more efficient than l-Glutamine alone. Oxidative stress is generated during the inflammation, particularly by the induction of nitrite oxide synthesis, and partly responsible for the mucosal damage associated to chronic inflammation. Attenuation of oxidative stress is therefore of interest in such cases.

The use of a co-culture model in our investigation offers a transformative approach to understanding the multifaceted effects of dietary constituents and their combinations. Unlike monoculture systems, the co-culture model more closely mimics the intricate interactions present in the gastrointestinal environment. This is pivotal in comprehensively elucidating the effects of diet on gut health, especially since dietary components can exert multiple modes of action simultaneously. For instance, certain dietary elements may influence mucus formation, thereby impacting the gut's protective barrier and its defense against pathogens. Simultaneously, they could modulate the integrity of tight junctions, which plays a cardinal role in maintaining gut permeability and preventing the translocation of unwanted substances. Furthermore, the immunomodulatory effects of dietary components underscore their potential in shaping immune responses, which can either benefit gut health or lead to inflammation and disease. Therefore, our co-culture model stands as an efficient tool, enabling investigations into these mechanisms and offering insights that could lead to the formulation of more effective dietary strategies for optimizing gut health [11]. Of course, our preliminary study presents some limitations, and more particularly needs to be confirmed *in vivo*, in humans or in animal models, in order to consider important factors contributing to the intestinal barrier function (*i.e.* microbiota, variety of immune cell types, 3D structure of the mucosae and enteric nervous system). Future experiments will permit to identify the different pathologies where researchers could compare the impact of PERMEA *vs* one food complement alone. In addition, *in vivo* approaches will be needed to identify the potential curative and/or protective action of PERMEA in pathologies associated with alteration of gut barrier, and/or intestinal microbiota [28].

To conclude, the present study showed multiple beneficial effects of the food supplement PERMEA on several parameters implicated in the leaky-gut syndrome: excessive transepithelial passage, inflammation and oxidative stress. This effect could not be attributed to l-glutamine alone, but rather to the combination of PERMEA's constituents. The clinical relevance of such results needs to be addressed.

## Funding statement

This research was funded by LABORATOIRE LESCUYER, Aytré, France.

## Data availability statement

Data will be made available on request.

## Ethics statement

Informed consent was obtained from all subjects involved in the study. Human cells were obtained from STEMCELL Technologies with the authorization #IE-2020-1126 and approved by the “WIRB-Copernicus Group (WCG) Institutional Review Board” ethic comity.

## CRediT authorship contribution statement

**Anne Abot:** Writing – review & editing, Writing – original draft, Validation, Supervision, Project administration, Investigation, Formal analysis, Conceptualization. **Nicolas Pomié:** Investigation, Formal analysis. **Gwendoline Astre:** Writing – review & editing, Supervision, Investigation, Formal analysis. **Patrice D. Cani:** Writing – review & editing, Writing – original draft, Formal analysis, Conceptualization. **Justine Aussant:** Writing – review & editing, Methodology, Formal analysis. **Emmanuel Barrat:** Writing – review & editing, Writing – original draft, Validation, Supervision, Project administration, Funding acquisition, Formal analysis. **Claude Knauf:** Writing – review & editing, Writing – original draft, Validation, Formal analysis, Conceptualization.

## Declaration of competing interest

PDC and CK are co-founders of Enterosys SAS (France), AA, NP and GA are employed by Enterosys SAS (France), EB and JA are employed by Laboratoire Lescuyer (France). PDC was co-founder of The Akkermansia company SA and coinventor on patents dealing with gut microbes and health. Laboratoire Lescuyer funded this independent study conducted by Enterosys S.A.S. (Labège, France) to investigate the impact of PERMEA on gut permeability. Enterosys is a Contract Research Organization (CRO) with no conflicting interests involving Laboratoire Lescuyer.
