# Supplementary material for: Effect of the dietary supplement PERMEAPROTECT+ TOLERANCE© on gut permeability in a human co-culture epithelial and immune cells model
Source: Heliyon. 2024 Mar 27;10(7):e28320. doi: 10.1016/j.heliyon.2024.e28320 (PMC10998107; doi:10.1016/j.heliyon.2024.e28320)
Supplement: Multimedia component 1 [file mmc1.docx]

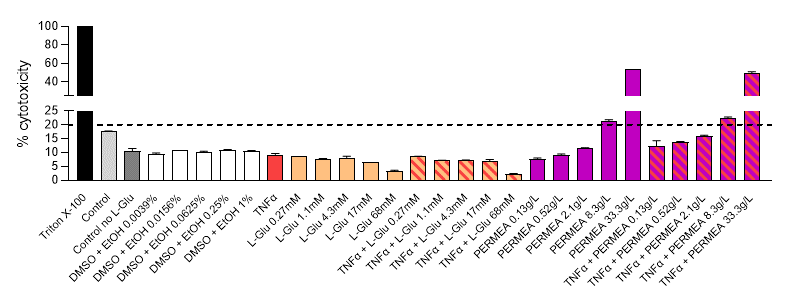


**Supplemental Figure 1:** Evaluation of LDH released by IEC. The results were expressed in percentage of the positive control n=2/group.


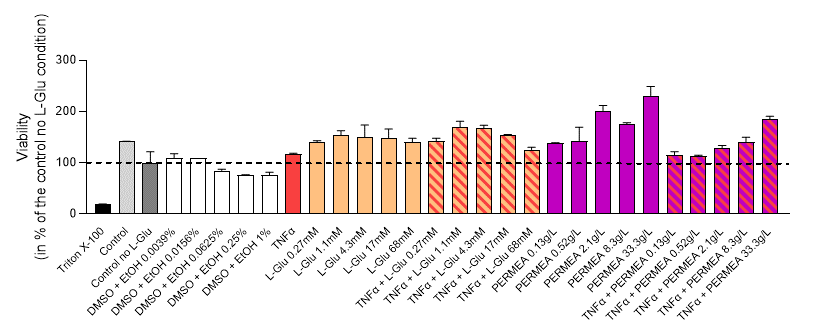


**Supplemental Figure 2:** Evaluation formazan production by IEC. The results were expressed in percentage of the control. The results were expressed in percentage of the positive control n=2/group.
