# Supplementary figures and images for: Effect of the dietary supplement PERMEAPROTECT+ TOLERANCE© on gut permeability in a human co-culture epithelial and immune cells model
Source: Heliyon. 2024 Mar 27;10(7):e28320. doi: 10.1016/j.heliyon.2024.e28320 (PMC10998107; doi:10.1016/j.heliyon.2024.e28320)

**Supplemental Table 1.** Characteristics of the PBMC donors involved in the study.


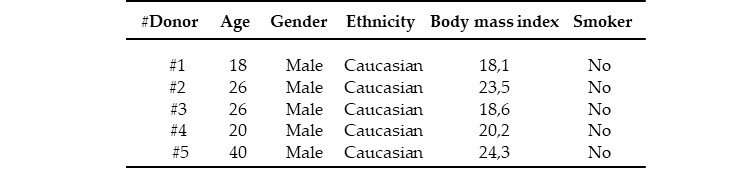

Supplement: Multimedia component 2 [file mmc2.docx]
