# Supplementary material for: Effect of the dietary supplement PERMEAPROTECT+ TOLERANCE© on gut permeability in a human co-culture epithelial and immune cells model
Source: Heliyon. 2024 Mar 27;10(7):e28320. doi: 10.1016/j.heliyon.2024.e28320 (PMC10998107; doi:10.1016/j.heliyon.2024.e28320)
Supplement: Multimedia component 3 [file mmc3.docx]

**Supplemental Table 2. Tested doses of PERMEA.**

The tested doses of PERMEA were chosen according to previous data provided by the sponsor and presented on Table 2.

| Bioactive Compounds | Dose previously tested | D1  1/1  (//dose in a glass) | D2  1/4  (//dose in the stomach) | D3  1/16 | D4  1/64 | D5  1/256 |
| --- | --- | --- | --- | --- | --- | --- |
| L-Glu | 10 mM  (2-40 mM) | 68 mM | 17 mM | 4,25 mM | 1,1 mM | 0.27 mM |
| PEA | 10 µM | 3.3 mM | 0.83 mM | 206 µM | 52 µM | 13 µM |
| Polyphenol | - | 4.7 mM | 1.2 mM | 294 µM | 73 µM | 18 µM |
| β-carotene | - | 9.3 µM | 2.3 µM | 581 nM | 145 nM | 36 nM |
| Vitamin D3 | 1-10 nM | 173 nM | 43.25 nM | 10.8 nM | 2.7 nM | 0.7 nM |
| Vitamin B1 | - | 18 µM | 4.5 µM | 1.1 µM | 281 nM | 70 nM |
| Vitamin B9 | - | 2.3 µM | 575 nM | 144 nM | 36 nM | 9 nM |
| Zinc | - | 765 µM | 191 µM | 47.8 µM | 12 µM | 3 µM |
| Final concentration | - | **33.3 g/L** | **8.3 g/L** | **2.1 g/L** | **0.52 g/L** | **0.13 g/L** |

*Table 2.a: Concentration of the different bioactive compounds in PERMEA solutions tested for cytotoxicity.*

| Bioactive Compounds | Dose previously tested | PERMEA X2 1/8 = ½ dose in the stomach | PERMEA X1 = D3 (1/16) | L-glutamine (reference) |
| --- | --- | --- | --- | --- |
| L-Glu | 10 mM  (2-40 mM) | 8,5 mM | 4,25 mM | 8,5 mM |
| PEA | 10 µM | 412 µM | 206 µM | - |
| Polyphenol | - | 588 µM | 294 µM | - |
| β-carotene | - | 1’162 nM | 581 nM | - |
| Vitamin D3 | 1-10 nM | 21.6 nM | 10.8 nM | - |
| Vitamin B1 | - | 2.2 µM | 1.1 µM | - |
| Vitamin B9 | - | 288 nM | 144 nM | - |
| Zinc | - | 95.6 µM | 47.8 µM | - |
| Final concentration | - | **4.2 g/L** | **2.1 g/L** | **8,5 mM** |

*Table 2.b: Concentration of the different bioactive compounds in the reference and PERMEA solutions tested in the final experiment.*
